# Unsupervised Clustering in Neurocritical Care: A Systematic Review

**DOI:** 10.1007/s12028-024-02140-w

**Published:** 2024-11-19

**Authors:** Jeanette Tas, Verena Rass, Bogdan-Andrei Ianosi, Anna Heidbreder, Melanie Bergmann, Raimund Helbok

**Affiliations:** 1 Department of Neurology, Kepler University Hospital, Johannes Kepler University Linz, Linz, Austria; 2 Clinical Research Institute for Neuroscience, Johannes Kepler University Linz, Linz, Austria; 3 Department of Neurology, Medical University of Innsbruck, Innsbruck, Austria

**Keywords:** Neurocritical care, Unsupervised clustering, Traumatic brain injury, Stroke, Hypoxic -ischemic brain injury, Neuromonitoring

## Abstract

Managing patients with acute brain injury in the neurocritical care (NCC) unit has become increasingly complex because of technological advances and increasing information derived from multiple data sources. Diverse data streams necessitate innovative approaches for clinicians to understand interactions between recorded variables. Unsupervised clustering integrates different data streams and could be supportive. Here, we provide a systematic review on the use of unsupervised clustering using NCC data. The primary objective was to provide an overview of clustering applications in NCC studies. As a secondary objective, we discuss considerations for future NCC studies. Databases (Medline, Scopus, Web of Science) were searched for unsupervised clustering in acute brain injury studies including traumatic brain injury (TBI), subarachnoid hemorrhage, intracerebral hemorrhage, acute ischemic stroke, and hypoxic-ischemic brain injury published until  March 13th 2024. We performed the systematic review in accordance with the Preferred Reporting Items for Systematic reviews and Meta-Analyses guidelines. We identified 18 studies that used unsupervised clustering in NCC. Predominantly, studies focused on patients with TBI (12 of 18 studies). Multiple research questions used a variety of resource data, including demographics,  clinical- and monitoring data, of which intracranial pressure was most often included (8 of 18 studies). Studies also covered various clustering methods, both traditional methods (e.g., k-means) and advanced methods, which are able to retain the temporal aspect. Finally, unsupervised clustering identified novel phenotypes for clinical outcomes in 9 of 12 studies. Unsupervised clustering can be used to phenotype NCC patients, especially patients with TBI, in diverse disease stages and identify clusters that may be used for prognostication. Despite the need for validation studies, this methodology could help to improve outcome prediction models, diagnostics, and understanding of pathophysiology.

*Registration number*: PROSPERO: CRD4202347097676.

**Supplementary Information:**

The online version contains supplementary material available at 10.1007/s12028-024-02140-w.

## Introduction

Critical care physicians are challenged by the increasing amount of available data in the management of neurocritical care (NCC) patients. Data acquisition is no longer limited to the recording of classic biosignals (temperature, blood pressure, intracranial pressure, etc.) but is constantly expanding. It now enables real-time collection and quantification of signal derived measures on hemodynamics, metabolic changes, and neuronal activity in an individual patient. Adequate bedside tools to integrate and visualize the complexity of diverse data streams to phenotype patients, are still lacking but should ideally be used in the future to adapt treatments. So far, clustering and machine learning techniques have been retrospectively applied to phenotype (i.e., based on observable characteristics) or endotype (i.e., related to different biological mechanisms) patients.

Clustering can be supervised, unsupervised, and semisupervised. The advantage of unsupervised clustering over supervised clustering is that it assigns data into clusters that do not require predefined labels or information, which characterizes supervised clustering. For semisupervised clustering, labels are used to guide the unsupervised clustering [1]. Hence, unsupervised clustering is the only method without guidance of the output data. However, unsupervised clustering can be challenging because there is commonly no ground truth against which results can be compared, and thus no consensus is available on the configuration of the clustering, including the optimal clustering algorithm, the number of clusters, and how to handle outliers in the data [2].

Unsupervised clustering can roughly be divided into five methods: partitioning, hierarchical, density-based, grid-based [3–5], and advanced model-based methods [3], but combined methods within one analysis are often seen. Briefly, partitioning clustering is characterized by the separation of data points based on the distance to predefined cluster centers; hierarchical clustering is organized as a hierarchical ‘tree’ showing the different numbers of clusters and connections; density-based clustering results can have multiple shapes; grid-based methods basically divide the data in multidimensional grids in which the density of data within grid cells differentiates clusters; and model-based methods use probability functions to allocate data to a clustering group [3].

The choice of a particular method depends on the characteristics of the data and the research question. Considerations for a particular method are that partitioning and density-based clustering are used only for numerical data, whereas hierarchical and model-based methods can handle different data formats. In addition, partitioning and density-based methods require a preselection of the number of clusters, so depending on this decision, the results are likely to be different. Another advantage of density-based methods over partitioning, hierarchical, and grid-based methods is that density-based methods are not restricted to finding a particular shape for the clusters (spherical or a grid) but also find s-shaped clusters, for example [3–5]. Because of the limitations of each method, a combination and novel unsupervised methods have been explored [5].

Unsupervised clustering has been applied in critical care medicine in several areas, and disease entities, such as acute respiratory distress syndrome [6], out-of-hospital cardiac arrest [7], COVID-19 [8, 9], sepsis [10, 11], and other diseases. In this line, phenotypes and their association with i.e., outcomes have been studied using unsupervised clustering with the aim to earlier identify high-risk patients, personalize ICU management and treatments, and select patients for future clinical trials [12]. However, the applicability and significance of data-driven unsupervised clustering for NCC patients is unknown. Here, we provide a systematic review of studies applying unsupervised clustering in NCC patients, with the aim of giving a bird’s eye overview, and discuss considerations for future NCC studies.

## Methods

We performed a systematic review in accordance with the Preferred Reporting Items for Systematic reviews and Meta-Analyses [13] (Supplementary Table 1). The protocol was registered in the international prospective register of systematic reviews (PROSPERO: CRD4202347097676).

### Eligibility Criteria

For the search, we included studies including (1) patients admitted to the intensive care unit (ICU) across all ages; (2) patients with a primary neurological disease, including traumatic brain injury (TBI), subarachnoid hemorrhage (SAH), intracerebral hemorrhage (ICH), acute ischemic stroke (AIS), and hypoxic-ischemic brain injury (HIBI); and (3) study aims and/or objectives related to a data clustering method on a patient level (i.e., ICU data must have been part of the data set). Studies including patients with a secondary diagnosis of brain injury were excluded from the analysis (e.g., COVID-19, sepsis). In addition, reviews, meta-analysis, and study protocols were excluded.

### Study Objectives

The primary objective was to provide an overview of unsupervised clustering applications in NCC populations. The secondary objective was to discuss considerations for future NCC studies.

### Search Strategy

We searched the online platforms Medline (PubMed), Elsevier (Scopus), and Clarivate (Web of Science) for unsupervised clustering in NCC patients, with the search terms “clustering analysis”, “intensive care,” “traumatic brain injury”, “subarachnoid hemorrhage”, “intracerebral hemorrhage”, “acute ischemic stroke”, and “hypoxic-ischemic brain injury”. Our initial search was conducted on July 14, 2023, and updated on March 13, 2024. The search strings are available in Supplementary Table 2.

### Study Selection and Data Extraction

Two independent reviewers (JT, VR) screened the abstracts according to the inclusion and exclusion criteria. The results were discussed until consensus was reached, with the possibility to discuss with a third independent reviewer. After full-text reading and study selection, the abstracts of references and citations of the selected studies were again screened for additional studies and discussed for inclusion among the reviewers. The management software Citavi (Swiss Academic Software, Citavi, computer software, 2023) was used for abstract screening. Thereafter, one reviewer extracted various study variables. This was verified by the second reviewer. The following variables were extracted from each study: author, journal, Epub year, study type, title, diagnosis, sample size, sex, age, study period, data period, sample frequency of the clustering data, study aim, data used for clustering, clustering method, number of clusters and how this number was determined, distance metrics, handling of missing data/artifacts, validation method, analysis software, main outcome measures, functional outcome assessed and result, main results, and conclusion (Supplementary file 2). Thereafter, we summarized the study characteristics and the results in tables and an alluvial plot (R-packages ggplot [14] and ggalluvial [15]). The summarized results were used to formulate considerations for future NCC studies.

### Risk of Bias Assessment

In the absence of an established bias ranking for clustering algorithms, a systematic, objective, and reproducible assessment could not be performed. In addition, we attempted to obtain missing data by contacting the authors or reporting it as missing if not available.

## Results

**Fig. 1:**
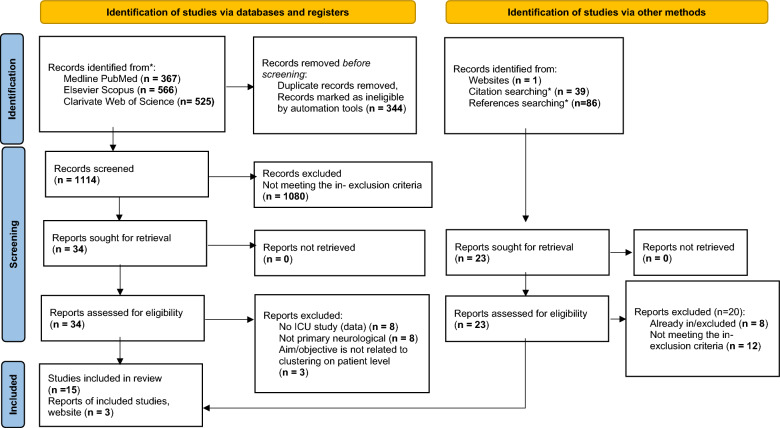
Study flowchart adopted from Page et al. [13]. *The citations and references were automatically filtered using the search string: [(Clustering OR Cluster OR Pattern OR Clustering OR Phenotype) AND (""Intensive care" OR "critical care" OR ICU) AND (brain OR neuro OR subarachnoid OR SAH OR ICH OR "ischemic stroke" OR "cardiac arrest" OR cerebral)]. Thereafter the abstracts were screened, and eligible studies were selected

Of 1,114 studies, 72 studies were selected by the two reviewers. After discussion, 34 studies were selected for full-text reading. Nineteen additional studies were then excluded, of which eight studies appeared to meet the inclusion criteria but were excluded because of lack of data collected in the ICU [16–23]. With the inclusion of three studies identified from other methods, 18 studies met all inclusion criteria (Fig. 1), with 5 of 18 studies including (primarily) pediatric patients. The average number of patients included in the 18 selected studies was 42 (interquartile range 23–313). The majority focused on TBI (12 of 18 studies), followed by five studies that included diverse combinations of populations (TBI, SAH, ICH, AIS), and only one study included patients with HIBI. Most studies (9 of 18 studies) were published in 2020 or later. A summary of the study characteristics is presented in Table 1. The individual study aims, clustering data, and outcome measures are listed in Table 2.

**Table 1:** Study characteristics, patient cohorts, and methods used

	Number of studies (*N* = 18)
Diagnosis	
Traumatic brain injury	12 (67%)
Hypoxic-ischemic brain injury	1 (5.6%)
Diverse patient populations^a^	5 (28%)
Male%, median (IQR)	73 (54–78)
Patient age range^b^	
0–1 y	1 (5.9%)
2–18 y	3 (18%)
19–50 y	8 (47%)
51–70 y	5 (29%)
Sample size, median (IQR)	42 (23–313)
Year of publication	
2004–2008	3 (17%)
2009–2012	2 (11%)
2013–2016	1 (5.6%)
2017–2020	3 (17%)
2021 to March 13th 2024	9 (50%)
Study period^c^	
0–1 y	2 (15%)
2–4 y	6 (46%)
5–10 y	3 (23%)
> 10 y	2 (15%)
Data collection period	
0–1 h	1 (5.6%)
2–24 h	1 (5.6%)
> 1 d	11 (61%)
A single measurement point^d^	5 (28%)
Unsupervised clustering method^e^	
Hierarchical	10 (56%)
K-means	6 (33%)
GBTM	1 (5.6%)
Bayesian approach	2 (11%)
MOCAIP	2 (11%)
Hidden Markov models	1 (5.6%)
Spectral clustering	1 (5.6%)
Kohonen SOM	1 (5.6%)

*AIS* Acute ischemic stroke, *GBTM* Group-based trajectory modeling, *ICH* Intracerebral hemorrhage, *IQR* Interquartile range, *MOCAIP* Morphological clustering and analysis of intracranial pressure, *SAH* Subarachnoid hemorrhage, *SOM* Self-organizing maps, *TBI* Traumatic brain injury

^a^The included diagnosis were TBI (3 studies), SAH (5 studies), ICH (4 studies), and AIS (1 study)

^b^In 1 study, the age was not reported

^c^Duration of the study as reported by the authors. In 5 studies, the study period was not reported

^d^Example of a single measurement point means demographic data

^e^Several studies apply a combination of 2 or more clustering techniques or apply 2 or more separate clustering techniques

**Table 2:** Individual unsupervised clustering studies (*N* = 18)

Study	Dx	Sample	Aim	Unsupervised clustering method	Clustering data	Outcome measure
Nelson et al. [32]	TBI	26	To examine CMD patterns in TBI patients and explore their relationships with ICP and CPP using neural network methodology	Kohonen SOM	Hourly CMD samples	Relationship with ICP, CPP, GOS
Haqqani et al. [40]	TBI	6 (pediatrics)	To validate gel-free proteomic methods for identifying peripheral "surrogate markers" of brain injury using k-means clustering. And to assess the utility of pattern analyses, such as hierarchical clustering, in recognizing common behavior among proteins of peripheral or brain origin compared to established brain injury biomarkers like S100B or clinical injury classifiers	k-means, hierarchical	Blood samples (serum proteins)	Identification of brain biomarker patterns to gain information on disease characteristics in comparison to a single marker or injury severity (GCS)
Sorani et al. [34]	TBI	23	To describe challenges and opportunities of high-frequency multimodality monitoring data for 1) the quantification of secondary brain insults, 2) development of clustering methodology to construct “profiles” for diagnosis and treatment	Hierarchical	1-min (neuro-) monitoring data and ventilatory data	Exploration of clusters
Kim et al. [37]	TBI (29), SAH (15), ICH (2), NPH (1)	47	To extend the application of the MOCAIP algorithm for extracting diverse morphological metrics from simultaneous recordings of ICP and CBFV pulses in brain injury patients and explore the correlations among these metrics	MOCAIP (includes hierarchical clustering)	ICP waveform, CBFV waveform	Interrelationships among the morphological metrics (extracted through clustering) of the ICP and CBFV waveforms
Wainwright et al. [29]	TBI (12), ICH (7), stroke (4), DKA (1), ADEM (1), ALF (5), metabolic (1)	31 (pediatrics)	To analyze a large data set of heterogeneous data classes (laboratory, physiology, device settings, socioeconomic status), collected as part of the routine care of children with acute brain injuries who underwent ICP monitoring	Agglomerative hierarchical	Laboratory, physiology, device settings, socioeconomic status	Clustering results in comparison with different outcome groups
Kumar et al. [41]	TBI	114	To apply PCA on CSF inflammatory marker data obtained for dimension reduction. The PCAs were used to identify meaningful clusters within the study population. Clusters were compared with other (CSF) biomarkers and clinical outcome	k-means	CSF samples (proteins)	GOS
Jha et al. [27]	TBI	404	To characterize longitudinal ICP trajectories and discern potential distinctive patterns in severe TBI	GBTM	ICP waveforms	GOS, DRS
Asgari et al. [28]	TBI	379	To develop a method for the detection of hourly physiological states and relate these to the clinical outcome	Hidden Markov models	Hourly neuromonitoring data (ICP, CPP, PRx, RAP)	GOS
Eiden et al. [39]	TBI	38	To combine metabolomics with data modeling and in silico pathway analysis to define metabolic states	Hierarchical	Hourly CMD samples	Identification of metabolic states, association with GOS/TIL
Gradisek et al. [36]	TBI	80	To identify patterns of circulating biomarkers and associate them with the GOSE	Hierarchical	Blood samples (serum proteins)	GOSE, 14-day mortality
Lindblad et al. [26]	TBI (90), healthy (15)	105	To screen for inflammatory proteins in blood and CSF and to assess their role in BBB disruption, neuroinflammation and long-term outcome	Hierarchical	CSF and blood samples (proteins)	Protein concentrations in relation to BBB disruptions and GOS
Megjhani et al. [35]	ICH, SAH, brain tumor	19	To examine whether changes in ICP waveform morphologies can be used as a biomarker for early detection of ventriculitis	MOCAIP (includes hierarchical clustering), k-means	ICP waveform	Comparison of the distribution of the meta clusters before, during, and after ventriculitis
Narula et al. [30]	SAH (19), EDH (1), ICH (2), epilepsy (8)	29	To describe a novel unsupervised learning algorithm that detects bursts in EEG and generate burst per minute estimate. In addition, validate the algorithm on annotated EEG data	Spectral clustering, k-means	EEG waveform	Comparison of the unsupervised with manually labeled and supervised methodology
Åkerlund et al. [25]	TBI	1728	To describe distinct endotypes based on admission variables and to assess their association with clinical outcomes	Probabilistic Graph Model (Bayesian Approach)	Demographics, initial assessment data (< 24 h after ICU admission)	Description of 7 endotypes, association of endotypes with GOSE
Boos et al. [38]	TBI	500 (pediatrics)	To cluster patient data by 3 clustering methods and compare with the clinical or predicted diagnosis of abusive head injury	k-means, divisive/agglomerative hierarchical	Patient history, clinical data, initial assessment	Relationship between clusters and the clinical diagnosis of abusive head injury, and the diagnosis based on the triad (based on known predictors)
Rajagopalan et al. [33]	TBI (14), SAH (6)	20	To evaluate a data driven approach for the identification of physiological states	Agglomerative hierarchical	Hourly (neuro-) monitoring data (CMD, ICP, PbtO_2_, HR, MAP)	Relationship with outcome (defined as discharge location)
Satar et al. [31]	HIBI	15 HIBI, 20 healthy (pediatrics)	To investigate whether crying sounds can be used in the early diagnosis of HIBI for developing a computer-assisted device or app	k-means	Computed features from sound data	The best feature combination to detect HIBI compared to the clinical diagnosis
Åkerlund et al. [24]	TBI	1,728	To identify clinical variables that might distinguish disease trajectories among patients with traumatic brain injury admitted to the ICU	Probabilistic graph model (Bayesian approach) with a Markov chain extension	Clinical and laboratory variables	Description of trajectories during ICU stay

Studies are listed by their EPUB publication year. The numbers between brackets refers to the patient numbers per group. Sample refers to the sample size/number of included patients

*ADEM* Acute disseminated encephalomyelitis, *ALF* Acute liver failure, *BBB* Blood–brain barrier, *CBFV* Cerebral blood flow velocity, *CMD* Cerebral microdialysis, *CPP* Cerebral perfusion pressure, *CSF* cerebrospinal fluid, *DKA* Diabetic ketoacidosis, *DRS* Disability rating score, *Dx* Diagnosis, *EDH* Epidural hemorrhage, *EEG* Electroencephalogram, *GBTM* Group-based trajectory modeling, *GCS* Glasgow Coma Scale, *GOS* Glasgow Outcome Scale, *GOSE* Glasgow Outcome Scale-Extended, *HIBI* Hypoxic-ischemic brain injury, *HR* Heart rate, *ICH* Intracerebral hemorrhage, *ICP* Intracranial pressure, *ICU* Intensive care unit, *MAP* Mean arterial pressure, *MOCAIP* Morphological clustering and analysis of intracranial pressure, *NPH* Normal pressure hydrocephalus, *PbtO*_*2*_ Brain tissue oxygen tension, *PCA* principal component analysis, *PRx* pressure reactivity index, *RAP* The correlation coefficient (*R*) between mean pulse amplitude and mean intracranial pressure, *SAH* Subarachnoid hemorrhage, *SOM* Self-organizing maps, *S100B* S100 calcium-binding protein B,  *TBI* Traumatic brain injury, *TIL* Therapy intensity level scale

### Clustering Data

**Fig. 2:**
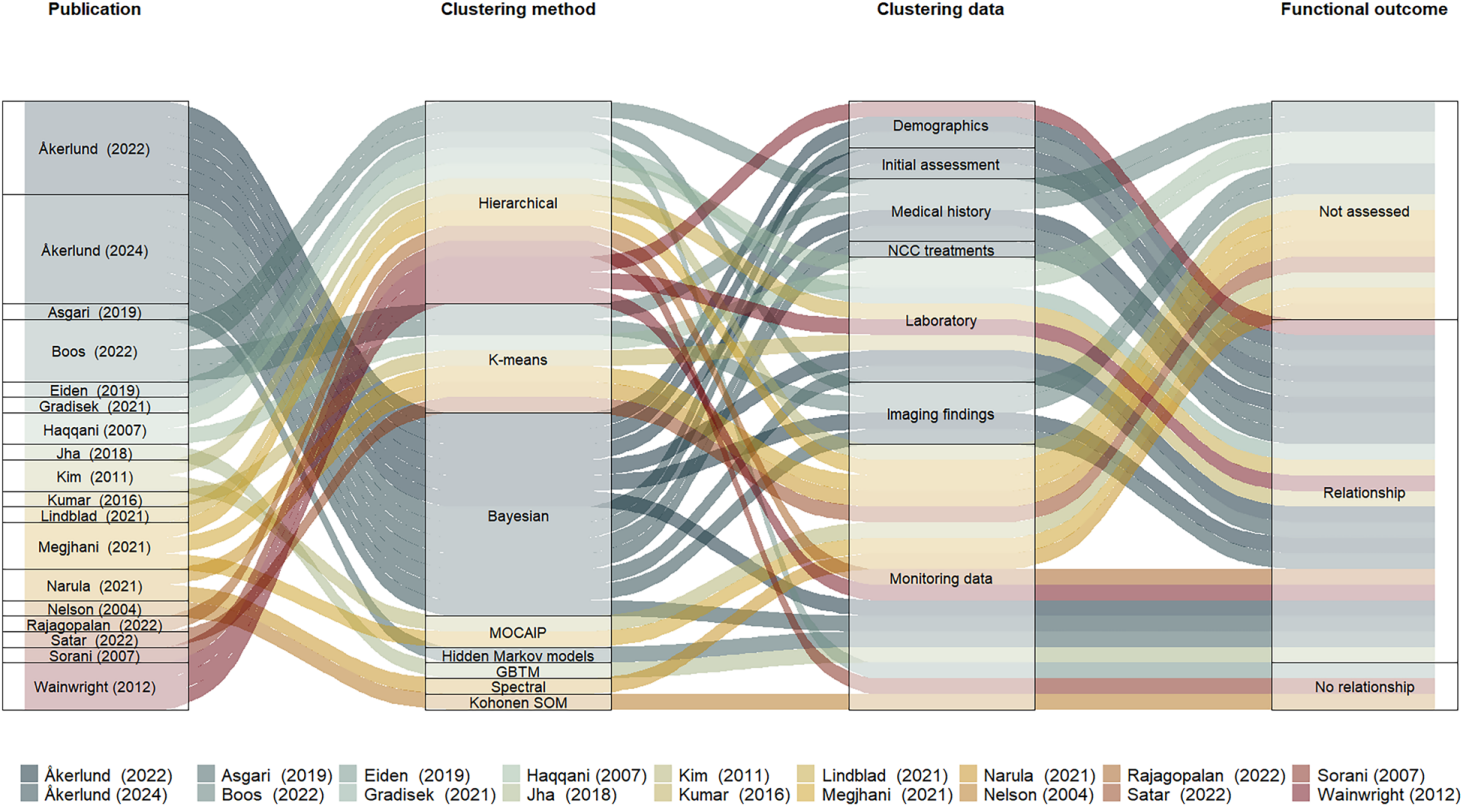
An overview of the information flow between studies (*N* = 18), the corresponding unsupervised clustering methods, and the clustering data. In the alluvial plot’s final column, functional outcome results are shown assessed by the Glasgow Outcome Scale-Extended, Disability Rating Score, or mortality. The publications are listed alphabetically (first author), the unsupervised clustering methods according to the frequency of occurrence, the clustering data by data type. Three methods shown are variations of probabilistic graph model approaches: Bayesian models, GBTM and unsupervised hidden Markov model. The Bayesian models include both a standard model and an extension incorporating Markov chain extension. The monitoring data (and derived measures) included: microdialysis, audio data, intracranial pressure, cerebral perfusion pressure, pressure reactivity index, the correlation coefficient (*R*) between mean pulse amplitude and mean intracranial pressure, arterial blood pressure, heart rate, cerebral/body temperature, brain tissue oxygen partial pressure, cerebral blood flow velocity, peripheral oxygen saturation, electroencephalography. Functional outcomes are listed sequentially, the studies that did not assess functional outcome (not assessed), the presence (relationship) or absence (no relationship) of a significant difference between the clustering results. The size of the individual bars corresponds to the number of in/output flows and may not correlate with the number of patients/methods included in the individual studies; *GBTM* Group-based trajectory modeling, *MH* Medical history, *MOCAIP* Morphological clustering and analysis of intracranial pressure, *NCC* Neurocritical care, *SOM* Self-organizing maps. A brief description of each method is available in the Supplementary Tables 3 and 3 and Supplementary Fig. 3

The alluvial plot (Fig. 2) illustrates the connections between unsupervised clustering methods, the data used for clustering, and information about functional outcomes of all studies included. Various data types were clustered: demographics, initial assessment, medical history, NCC treatments, laboratory values, imaging findings, and mostly monitoring data (included in 13 of 18 studies). Monitoring data was presented as waveforms, averaged values, as a single clustering input, or combined with other signals. Intracranial pressure (ICP) was the most commonly included variable (8 of 18 studies) within monitoring data. NCC treatments were only studied once and included ICP/cerebral perfusion pressure (CPP) therapies and the presence of a decompressive craniectomy [24].

The data quality within the studies was assessed by extracting how missing values or artifacts were handled within the studies. Most studies (14 of 18 studies) reported about handling missing values and/or artifacts with different approaches: data imputation [24–26], only analyzing data within clinical physiological ranges [27], excluding patients or variables with long data gaps [28] or many missing values [29], replacement of artifacts for white noise in electroencephalography (EEG) data [30], filtering noise in audio signals using a bandpass filter [31], excluding outliers/artifacts automatically or manually [32–34], and other methods [35–37]. Four studies did not report about handling missing values or artifacts [38–41].

### Variability of Research Questions of the Studies

The studies that applied unsupervised clustering in NCC patients are mainly exploratory with single or multiple objectives. Most studies were primarily interested in the ability to identify patterns and thus differences between the obtained groups based on their clustering data [24, 25, 28, 29, 32–34, 39]. To illustrate this, Asgari et al. [28] attempted to describe patients’ hourly states using averaged ICP data and derived measures. Eiden et al. [39] studied metabolic profiling of microdialysis (MD) samples and found relationships with routine clinical physiological markers and clinical outcomes. There were also studies specifically interested in finding new or surrogate biomarkers [27, 40]. For example, Jha et al. [27] studied differences in gene expression (*ABCC8* polymorphisms) among NCC patients as a potential new biomarker for describing the severity of TBI, as this gene is coding for a transmembrane protein that is also located in the brain, upregulated after injury, and plays a role in cerebral edema regulation [27, 42, 43]. Furthermore, authors of a recently published Collaborative European Neuro Trauma Effectiveness Research in TBI (CENTER-TBI) study identified brain biomarkers (S100 calcium-binding protein B (S100B), neuron-specific enolase (NSE), neurofilament light (NFL), tau, ubiquitin carboxy-terminal hydrolase L1 (UCH-L1), and glial fibrillary acidic protein (GFAP)) and glucose variability as major determinants of 12 different disease trajectories after TBI within the first week after ICU admission [24]. A third question type was clustering data for the development or extension of a ‘stand-alone’ algorithm. Narula et al. [30] developed an automated detection algorithm using EEG signals to detect burst suppression. Finally, clustering was used to determine prognostic markers for clinical outcome [24–29, 32–34, 36, 39, 41] (see Determination of prognostic markers for clinical outcome section for more details).

### Clustering Methods

Eight different clustering methods were applied either alone or in combination: hierarchical clustering, k-means, spectral clustering, Kohonen self-organizing maps, morphological clustering and analysis of intracranial pressure (MOCAIP), and three methods that are a mixture of probabilistic graph model approaches: a Bayesian model, unsupervised hidden Markov models, and group-based trajectory modeling (GBTM). A concise description of each method is given in Supplementary Tables 3 and 3 and Supplementary Fig. 3.

The majority of studies applied the traditional methods of hierarchical clustering (10 of 18 studies) and k-means (6 of 18 studies), which were primarily hypothesis generating. Eight studies applied novel analytical methods in combination with or without traditional approaches. Most of these methods (5 of 8 studies) were investigated in the last 5 years and address the limitations of traditional methods, such as preserving the temporal aspect within the data. This allows comparison of patients with similar time trajectories during the NCC stay [27]. For example, Jha et al. [27] used GBTM. They clustered patients based on similar ICP trajectories and investigated the relationship of these obtained clusters with other clinical variables and found six ICP trajectory groups. Comparison of patients within the trajectories showed that age, presence of craniectomy, type of hemorrhage, and clinical outcome differed between these clusters [27]. In contrast, Asgari et al. [28] applied unsupervised hidden Markov models using ICP, CPP, pressure reactivity index (PRx), and the correlation coefficient (*R*) between mean pulse amplitude and mean intracranial pressure (RAP); they did not study patient trajectories, but rather determined hourly ‘states.’ Therefore, the different clustering groups may contain similar patients but different time periods. Comparison of the clusters (‘state’ groups) showed differences in mean ICP, CPP, PRx, RAP, and clinical outcome: the percentage of time spent in the ‘good’ state (i.e., the lowest ICP and a negative PRx), representative of intact cerebral autoregulation, was significantly lower in patients who died and was higher in those with a good outcome (Glasgow Outcome Scale score = 5) [28].

The MOCAIP algorithm is a combination of unsupervised clustering with advanced analyses. It was developed to extract meaningful features from ICP waveforms by using hierarchical clustering in two steps. First, ICP pulses were clustered and averaged (called the dominant ICP pulse). Second, the dominant pulses were clustered again and compared to a reference library of ICP pulses to remove artifactual ICP pulses [44]. Using the MOCAIP methodology, Megjhani et al. [35] clustered ICP features and demonstrated the ability to discriminate between patients with and patients without ventriculitis. Narula et al. [30] applied spectral clustering to detect burst suppression in EEG data combined with k-means to group data points with similar signatures. In this study, the authors were able to develop an algorithm that, after further validation, could be used as a tool to assess the coma depth in ICU patients.

### Determination of Prognostic Markers for Clinical Outcome

Most studies (12 of 18) compared the clinical outcome across the different clusters (Fig. 2). Disease phenotypes were associated with functional outcomes in 9 of 12 studies, assessed by the Glasgow Outcome Scale—Extended score, the Disability Rating Score, or mortality. Both expected and unexpected associations were found. Rajagopalan et al. [33] clustered monitoring data (ICP, mean arterial blood pressure [MAP], MD, brain tissue oxygenation [PbtO_2_], and heart rate) from 20 patients with TBI and SAH. Patients in the following clusters were more likely to have an unfavorable outcome, defined as in-hospital mortality or discharge to a chronic nursing facility: cluster 1, higher MAP and higher MD-lactate and MD-pyruvate; cluster 2, higher heart rate and MD-lactate/pyruvate ratio along with lower MAP, PbtO_2_, and MD-glucose. In comparison, patients in a cluster with a higher PbtO_2_ and MD-glucose combined with lower ICP had a more favorable outcome. They compared their results with a group-based analysis showing that the outcome differences were more pronounced in the clustering analysis [33]. Jha et al. [27] incorporated the temporal aspect of ICP by using GBTM and showed that patients with high ICP value trajectories, but also those with low ICP value trajectories, had poor outcomes. With this finding, they emphasize the importance of pattern recognition over single-point measurements/thresholds [27].

Finally, Åkerlund et al. [25] reported a clustering model that showed improved outcome prediction compared with the International Mission for Prognosis and Analysis of Clinical Trials in TBI (IMPACT) prediction score. They included data collected before ICU admission and early in the ICU stay. Significant features for their clustering model included the Glasgow Coma Scale (GCS) motor score, the GCS total score, lactate, oxygen saturation, creatinine, glucose, base excess, pH, partial pressure of carbon dioxide in arterial blood, and body temperature. The resulting clusters were incorporated in the IMPACT score and showed increased outcome prediction for both mortality and unfavorable outcome [25].

## Discussion

The primary objective was to provide an overview of clustering applications. The main finding is that most studies investigated TBI patient cohorts, and various resource data were clustered, of which ICP was the most used brain-derived monitoring variable in the analysis, suggesting that clustering methods were mostly used in patients with severe acute brain injury.

Furthermore, the ability to study various data types and formats (e.g., averaged data, waveforms) for clustering and comparing results allowed us to answer a range of research questions. However, to the best of our knowledge, there are no intervention studies based on clustering results. The ultimate goal in this context is that a particular combination of clinical (monitoring) variables will assign a patient to a particular treatment strategy aimed at optimizing cerebral blood flow, brain oxygenation, and substrate delivery to the brain and integrate appropriate management of systemic complications to prevent secondary brain injury and improve outcomes in NCC patients.

Both traditional and advanced methods were studied. Especially, the advanced clustering methods allow for the inclusion of longitudinal changes in physiological and clinical parameters, which is particularly important for delineating disease trajectories and can add valuable information to admission data. Additionally, the use of advanced methods based on a combination of traditional and novel methods in unsupervised clustering allows for the development of stand-alone methodologies with direct applications in the clinical practice [30, 35, 37]. In particular, the aggregation of different variables into biologically meaningful states and trajectories might have the potential to characterize patient phenotypes and endotypes to improve individualized medicine, as mentioned by Jha et al. [27].

Finally, studies found new associations between distinct resource data, and in most studies, authors were able to differentiate outcome groups based on cluster results. In a retrospective approach, data collected during the acute phase after TBI were associated with long-term functional outcomes [25]. The clustering model even outperformed the traditional IMPACT outcome prediction score in patients with TBI.

### Considerations for the Use of Unsupervised Clustering in NCC

As a secondary objective, we discuss considerations for future NCC studies. Despite the promising results within the individual studies, unsupervised clustering methods and their applications in NCC have various gaps. These gaps need to be addressed to improve study quality, comparability, and interpretation of the results.

### Heterogeneity of Study Populations and Limitations in Sample Size

Five studies analyzed different diseases in their clustering analysis [29, 30, 33, 35, 37], which may mask phenotypes for specific patient groups and emphasize heterogeneity within studies. In addition, the median cohort size was 42 (interquartile range 23–313), with three studies including less than 20 patients [31, 35, 40]. For partitioning algorithms, Dalmaijer et al. [45] found that a minimum number of 20 patients per subgroup (cluster) were required to have sufficient statistical power for k-means. However, another group derived a formula to calculate the sample size that depends on the number of clusters and the number of variables [46].

### Multimodality Monitoring

The integration of multimodality neuromonitoring data in addition to ICP data was underrepresented. Only three studies included two or more neuromonitoring signals in their clustering analysis [33, 34, 37]. Especially in neurocritical patients, the integration of multiple neuromonitoring signals rather than single signals seems promising to discover new patterns directly at the organ of interest.

### Clustering Interpretation

Each clustering approach requires different parameters to be defined before the actual clustering starts. Although there are methods to assist in the selection of configurations, a degree of subjectivity is inherent in these configurations. The algorithms on its turn are robust and systematic, but the results and thus their interpretation carry the subjectivity of the configuration forward [47]. Nevertheless, if similar phenotypes are shown in validation cohorts, the results are more valid, and it indicates that the configuration of the parameters was effective, supporting the need for external validation cohorts when using clustering methods. In addition to methodological challenges, the clinical implementation of unsupervised clustering models comes with challenges such as the need for infrastructure restricted to dedicated ICUs. Although real-world clinical implementation for immediate adaptation of patient management, including early estimation of complications and clinical outcomes that would prompt treatment adjustments, is a future perspective, some approaches such as automatic detection of EEG burst suppression [30] may already be feasible in clinical practice.

### Clustering Evaluation

Comparison of unsupervised studies in terms of potential bias and quality assessment of clustering methods is difficult because of the lack of a well-established and systematic bias ranking template. This would be beneficial for both study quality and the ability to validate results. Kamber et al. [5] recommended reporting quality, stability, and tendency of the clustering methods/results when applying unsupervised clustering. Quality refers to how well the clustering results match with a ground truth (extrinsic validation) and/or assessment of the clusters (intrinsic validation). An example of extrinsic validation was given by Narula et al. [30], who studied an unsupervised clustering method for the detection of burst suppression. They compared their clustering results with annotated data from a neurologist. Clearly, this extrinsic validation is only possible if the expected result is known [30]. For intrinsic assessment, the different clustering settings are evaluated within a study. To illustrate, the silhouette coefficient was used by Boos et al. [38]. This coefficient ranges from − 1 to 1, with a preferred value of 1 meaning that the clusters are far apart [38]. In line with the quality assessment is the examination of stability. This includes determining the optimal number of clusters. Rajagopalan et al. [33] computed the Calinski–Harabasz pseudo F index to determine the optimal number of clusters. They computed this index for 2–10 clusters, with larger indices indicating clusters that are further apart [33]. Tendency refers to the (statistical) evaluation of whether unsupervised clustering is suitable for the data [5]. Other aspects to consider when analyzing and reporting results include reporting missing values, software packages and versions used, and reporting of cluster parameter settings [5]. Finally, although some algorithms can handle missing values, dedicated clinical care and thus data quality is the basis for avoiding significant bias and obtaining relevant results.

### Validation Studies

Only one study used a validation cohort [39], which challenges the significance of the results reported here, as unsupervised clustering requires several decisions that affects the results (i.e., preprocessing steps, clustering algorithm, number of clusters, number of variables, type of cluster assessment). Therefore, validation of the current results in different cohorts seems to be a necessary step before the study results can be interpreted and used for outcome prediction.

In the near future, unsupervised clustering may gain strength and novel insights by applying methods including various multimodal neuromonitoring data in large sample sizes for the entire population of NCC patients and may improve (the reporting of) clustering evaluations, which requires validation studies to confirm results.

### Limitations of the Systematic Review

Although our review provides insights into unsupervised clustering in NCC studies, we acknowledge the limitations of our review. We were interested in primary neurological patients admitted to the ICU, and therefore we excluded ICU patients in whom clustering analysis was performed with data not derived in the ICU. Therefore, there might be an underrepresentation of outcome prediction studies when they only used emergency department data. Furthermore, unsupervised clustering may have been applied as a preprocessing step for supervised machine learning, which was not the aim of the current analysis. Although we are aware of the (methodological) limitations of unsupervised clustering, in that there is no ground truth to compare the results obtained, and these results are themselves highly depended on the configuration decisions of the clustering method, we were still intrigued by how the methodological approach of unsupervised clustering impacted NCC and in what way it could guide future directions.

## Conclusions

Unsupervised clustering holds the possibility to endotype and phenotype diseases and disease stages in diverse NCC patient populations, especially those with TBI, by using distinct clustering data, including demographic, clinical, and monitoring data. Despite the need for validation studies, unsupervised clustering in NCC showed a range of applications and has the potential to help understand pathophysiology and improve prognostic approaches.

## Supplementary Information

Below is the link to the electronic supplementary material.

Electronic supplementary material (PDF 427 KB)

Electronic supplementary material (EXCEL 120 KB)
