# Supplementary material for: Unsupervised Clustering in Neurocritical Care: A Systematic Review
Source: Neurocrit Care. 2024 Nov 19;42(3):1074–86. doi: 10.1007/s12028-024-02140-w (PMC12137476; doi:10.1007/s12028-024-02140-w)
Supplement: Supplementary file 1 — Electronic supplementary material (PDF 427 KB) [file 12028_2024_2140_MOESM1_ESM.pdf]

Unsupervised Clustering in Neurocritical Care: A Systematic Review

Jeanette Tas[1,2]*, Verena Rass[3]*, Bogdan-Andrei Ianosi[1,2], Anna Heidbreder[1,2] Melanie Bergmann[1,2], Raimund Helbok[1,2]

*Contributed equally

[1] Department of Neurology, Kepler University Hospital, Johannes Kepler University Linz, Austria

[2] Research Institute for Neuroscience, Johannes Kepler University Linz, Linz, Austria

[3] Department of Neurology, Medical University of Innsbruck, Innsbruck, Austria

**Supplementary table 1 |** PRISMA checklist

**Supplementary table 2 |** Search strings

**Supplementary table 3 |** Concise description of unsupervised clustering methods employed in neurocritical care studies

**Supplementary table 4 |** Brief summary of feature selection, clustering parameters, validation, and distance metrics (N = 18)

**Supplementary figure 1 |** Example of the k-means algorithm

| Section and Topic | Item # | Checklist item | Location where item is reported |
|---|---|---|---|
| **TITLE** | | | |
| Title | 1 | Identify the report as a systematic review. | Titlepage |
| **ABSTRACT** | | | |
| Abstract | 2 | See the PRISMA 2020 for Abstracts checklist. | Supl. Page 5 |
| **INTRODUCTION** | | | |
| Rationale | 3 | Describe the rationale for the review in the context of existing knowledge. | Introduction |
| Objectives | 4 | Provide an explicit statement of the objective(s) or question(s) the review addresses. | Methods |
| **METHODS** | | | |
| Eligibility criteria | 5 | Specify the inclusion and exclusion criteria for the review and how studies were grouped for the syntheses. | Methods |
| Information sources | 6 | Specify all databases, registers, websites, organisations, reference lists and other sources searched or consulted to identify studies. Specify the date when each source was last searched or consulted. | Methods |
| Search strategy | 7 | Present the full search strategies for all databases, registers and websites, including any filters and limits used. | Methods |
| Selection process | 8 | Specify the methods used to decide whether a study met the inclusion criteria of the review, including how many reviewers screened each record and each report retrieved, whether they worked independently, and if applicable, details of automation tools used in the process. | Methods |
| Data collection process | 9 | Specify the methods used to collect data from reports, including how many reviewers collected data from each report, whether they worked independently, any processes for obtaining or confirming data from study investigators, and if applicable, details of automation tools used in the process. | Methods |
| Data items | 10a | List and define all outcomes for which data were sought. Specify whether all results that were compatible with each outcome domain in each study were sought (e.g. for all measures, time points, analyses), and if not, the methods used to decide which results to collect. | Methods |
| | 10b | List and define all other variables for which data were sought (e.g. participant and intervention characteristics, funding sources). Describe any assumptions made about any missing or unclear information. | Methods |
| Study risk of bias assessment | 11 | Specify the methods used to assess risk of bias in the included studies, including details of the tool(s) used, how many reviewers assessed each study and whether they worked independently, and if applicable, details of automation tools used in the process. | Methods |
| Effect measures | 12 | Specify for each outcome the effect measure(s) (e.g. risk ratio, mean difference) used in the synthesis or presentation of results. | no |
| Synthesis methods | 13a | Describe the processes used to decide which studies were eligible for each synthesis (e.g. tabulating the study intervention characteristics and comparing against the planned groups for each synthesis (item #5)). | Methods |
| | 13b | Describe any methods required to prepare the data for presentation or synthesis, such as handling of missing summary statistics, or data conversions. | Methods |
| | 13c | Describe any methods used to tabulate or visually display results of individual studies and syntheses. | Methods |
| | 13d | Describe any methods used to synthesize results and provide a rationale for the choice(s). If meta-analysis was performed, describe the model(s), method(s) to identify the presence and extent of statistical heterogeneity, and software package(s) used. | NA |
| | 13e | Describe any methods used to explore possible causes of heterogeneity among study results (e.g. subgroup analysis, meta-regression). | NA |
| | 13f | Describe any sensitivity analyses conducted to assess robustness of the synthesized results. | NA |
| Reporting bias | 14 | Describe any methods used to assess risk of bias due to missing results in a synthesis (arising from reporting biases). | Methods |

| Section and Topic | Item # | Checklist item | Location where item is reported |
|---|---|---|---|
| assessment | | | |
| Certainty assessment | 15 | Describe any methods used to assess certainty (or confidence) in the body of evidence for an outcome. | NA |
| **RESULTS** | | | |
| Study selection | 16a | Describe the results of the search and selection process, from the number of records identified in the search to the number of studies included in the review, ideally using a flow diagram. | Results |
| | 16b | Cite studies that might appear to meet the inclusion criteria, but which were excluded, and explain why they were excluded. | Results |
| Study characteristics | 17 | Cite each included study and present its characteristics. | Table 2 |
| Risk of bias in studies | 18 | Present assessments of risk of bias for each included study. | Methods |
| Results of individual studies | 19 | For all outcomes, present, for each study: (a) summary statistics for each group (where appropriate) and (b) an effect estimate and its precision (e.g. confidence/credible interval), ideally using structured tables or plots. | Table 1 |
| Results of syntheses | 20a | For each synthesis, briefly summarise the characteristics and risk of bias among contributing studies. | NA |
| | 20b | Present results of all statistical syntheses conducted. If meta-analysis was done, present for each the summary estimate and its precision (e.g. confidence/credible interval) and measures of statistical heterogeneity. If comparing groups, describe the direction of the effect. | NA |
| | 20c | Present results of all investigations of possible causes of heterogeneity among study results. | Results |
| | 20d | Present results of all sensitivity analyses conducted to assess the robustness of the synthesized results. | NA |
| Reporting biases | 21 | Present assessments of risk of bias due to missing results (arising from reporting biases) for each synthesis assessed. | NA |
| Certainty of evidence | 22 | Present assessments of certainty (or confidence) in the body of evidence for each outcome assessed. | NA |
| **DISCUSSION** | | | |
| Discussion | 23a | Provide a general interpretation of the results in the context of other evidence. | Discussion |
| | 23b | Discuss any limitations of the evidence included in the review. | Discussion |
| | 23c | Discuss any limitations of the review processes used. | Discussion |
| | 23d | Discuss implications of the results for practice, policy, and future research. | Discussion |
| **OTHER INFORMATION** | | | |
| Registration and protocol | 24a | Provide registration information for the review, including register name and registration number, or state that the review was not registered. | Methods |
| | 24b | Indicate where the review protocol can be accessed, or state that a protocol was not prepared. | Methods |
| | 24c | Describe and explain any amendments to information provided at registration or in the protocol. | Methods |
| Support | 25 | Describe sources of financial or non-financial support for the review, and the role of the funders or sponsors in the review. | Funding info |
| Competing interests | 26 | Declare any competing interests of review authors. | COI statement |
| Availability of | 27 | Report which of the following are publicly available and where they can be found: template data collection forms; data extracted from included | Suppl. Table |

| Section and Topic | Item # | Checklist item | Location where item is reported |
|---|---|---|---|
| data, code and other materials | | studies; data used for all analyses; analytic code; any other materials used in the review. | |

| ABSTRACT Section and Topic | Item # | Checklist item | Reported (Yes/No) |
|---|---|---|---|
| **TITLE** | | | |
| Title | 1 | Identify the report as a systematic review. | yes |
| **BACKGROUND** | | | |
| Objectives | 2 | Provide an explicit statement of the main objective(s) or question(s) the review addresses. | yes |
| **METHODS** | | | |
| Eligibility criteria | 3 | Specify the inclusion and exclusion criteria for the review. | |
| Information sources | 4 | Specify the information sources (e.g. databases, registers) used to identify studies and the date when each was last searched. | yes |
| Risk of bias | 5 | Specify the methods used to assess risk of bias in the included studies. | |
| Synthesis of results | 6 | Specify the methods used to present and synthesise results. | yes |
| **RESULTS** | | | |
| Included studies | 7 | Give the total number of included studies and participants and summarise relevant characteristics of studies. | yes |
| Synthesis of results | 8 | Present results for main outcomes, preferably indicating the number of included studies and participants for each. If meta-analysis was done, report the summary estimate and confidence/credible interval. If comparing groups, indicate the direction of the effect (i.e. which group is favoured). | yes |
| **DISCUSSION** | | | |
| Limitations of evidence | 9 | Provide a brief summary of the limitations of the evidence included in the review (e.g. study risk of bias, inconsistency and imprecision). | yes |
| Interpretation | 10 | Provide a general interpretation of the results and important implications. | yes |
| **OTHER** | | | |
| Funding | 11 | Specify the primary source of funding for the review. | NA |
| Registration | 12 | Provide the register name and registration number. | yes |

*From:* Page MJ, McKenzie JE, Bossuyt PM, Boutron I, Hoffmann TC, Mulrow CD, et al. The PRISMA 2020 statement: an updated guideline for reporting systematic reviews. BMJ 2021;372:n71. doi: 10.1136/bmj.n71. For more information, visit: http://www.prisma-statement.org/

**Supplemental table 2 | Search strings**

| | **Medline** | **Elsevier** | **Clarivate** |
|---|---|---|---|
| **Platform** | **PubMed** | **Scopus** | **Web of Science** |
| **Date** | 13.03.2024 | 13.03.2024 | 13.03.2024 |
| **Searches** | | | |
| **Search result** | **367** | **566** | **525** |
| **Filter** | Classical Article, Clinical Study, Clinical Trial, Clinical Trial, Phase I, Clinical Trial, Phase II, Clinical Trial, Phase III, Clinical Trial, Phase IV, Multicenter Study, Observational Study, Randomized Controlled Trial, Humans, English. | LIMIT-TO ( SUBJAREA , "NEUR" ) OR LIMIT-TO ( SUBJAREA , "COMP" ) ) AND ( LIMIT-TO ( DOCTYPE , "ar" ) ) AND ( LIMIT-TO ( LANGUAGE , "English" ) ) | **English** (Languages) and **Article** (Document Types) |
| **Diseases (traumatic brain injury, subarachnoid hemorrhage, intracerebral hemorrhage, acute ischemic stroke, hypoxic brain injury)** | tbi traumatic brain injury[MeSH Terms] OR Traumatic brain injury OR head* OR cranial trauma OR closed head injury OR brain trauma OR acquired brain injury OR skull fracture OR intracranial injury OR cerebral trauma OR neurotrauma OR tbi OR acute stroke[MeSH Terms] OR brain bleeding OR subarachnoid OR sah OR stroke OR subarachnoid hemorrhage[MeSH Terms] OR cerebral haemorrhage OR intracerebral haemorrhage[MeSH Terms] OR intracerebral bleed OR intracerebral haemorrhage OR intracerebral hemorrhage OR ich OR cardiac arrest[MeSH Terms] OR cardiac arrest OR anoxic brain injury OR hypoxic encephalopathy OR hypoxic brain OR cerebral infarction OR ischemic brain damage OR acute cerebral ischemia OR ischemic encephalopathy OR cerebral infarction[MeSH Terms] OR acute ischemic stroke OR neuro* OR cerebral* OR brain* | ( TITLE-ABS-KEY ( ( traumatic AND brain AND injury ) OR head* OR ( cranial AND trauma ) OR ( closed AND head AND injury ) OR ( brain AND trauma ) OR ( acquired AND brain AND injury ) OR ( skull AND fracture ) OR ( intracranial AND injury ) OR ( cerebral AND trauma ) OR neurotrauma OR tbi OR ( brain AND bleeding ) OR subarachnoid OR sah OR stroke OR ( intracerebral AND bleed ) OR ( intracerebral AND haemorrhage ) OR ( intracerebral AND hemorrhage ) OR ich OR ( cardiac AND arrest ) OR ( anoxic AND brain AND injury ) OR ( hypoxic AND encephalopathy ) OR ( hypoxic AND brain ) OR ( cerebral AND infarction ) OR ( ischemic AND brain AND damage ) OR ( acute AND cerebral AND ischemia ) OR ( ischemic AND encephalopathy ) OR ( acute AND ischemic AND stroke ) OR neuro* OR cerebral* OR brain* OR intracerebral OR hemorrhage ) ) | ALL=(traumatic brain injury[MeSH Terms] OR brain trauma OR acquired brain injury OR neurotrauma OR head trauma OR (subarachnoid hemorrhage [MeSH Terms]) OR subarachnoid OR sah OR acute stroke[MeSH Terms] OR intracerebral haemorrhage[MeSH Terms] OR ich OR (cardiac arrest[MeSH Terms]) OR “Cardiac arrest” OR anoxic brain injury OR hypoxic encephalopathy OR (acute cerebral ischemia [MeSH Terms]) OR "acute cerebral ischemia" OR neuro* OR cerebral* OR brain (All Fields) ) |
| **Critical care** | Critical care [MeSH Terms] OR ICU OR Intensive care OR Critical care OR Intensive care[MeSH Terms] | ( TITLE-ABS-KEY ( ( critical AND care ) OR icu OR ( intensive AND care ) ) ) | ALL=((Critical care [MeSH Terms] OR ICU OR (Intensive care) OR (Critical care) OR Intensive care[MeSH Terms])) |
| **Clustering** | "cluster analysis"[MeSH Terms] OR ("cluster"[All Fields] AND "analysis"[All Fields]) OR "cluster analysis"[All Fields] OR ("cluster analysis"[MeSH Terms] OR ("cluster"[All Fields] AND "analysis"[All Fields]) OR "cluster analysis"[All Fields] OR "clustering"[All Fields] OR "clusterings"[All Fields] OR "cluster"[All Fields] OR "cluster s"[All Fields] OR "clustered"[All Fields] OR "clusterization"[All Fields] OR "clusters"[All Fields]) OR "cluster analysis"[MeSH Terms]) OR "unsupervised" [All Fields] | ( TITLE-ABS-KEY ( ( cluster AND analysis ) OR clustering* OR unsupervised ) ) | ALL=((cluster analysis OR clustering* OR (cluster analysis[MeSH Terms]) OR unsupervised)) |
| **Excluding COVID** | COVID [MeSH Terms] OR COVID OR SARS-CoV-2 | AND NOT ( TITLE-ABS-KEY ( covid or  "sars-cov-2" OR covid-19)) | ALL=(( COVID OR "SARS-CoV-2" OR COVID-19)) |

| **Supplementary table 3 \| Concise description of unsupervised clustering methods employed in neurocritical care studies.** There is a wide range of methodological options for clustering data, with each method having various alternative approaches. Hence, the descriptions in this overview concentrate on their (main) application in the included studies. | |
|---|---|
| Hierarchical | Organizes data in a hierarchical or a 'tree' of clusters from small to high similarity between the clusters. The two main types are agglomerative and divisive clustering. For agglomerative clustering, the clustering starts with all datapoints that combine to different clusters during each iteration, whereas divisive clustering starts with one cluster and iteratively divides into more clusters [1]. |
| K-means | K-means computes the shortest distance from each data point to a centroid, representing the center of a cluster. Through an iterative process, the algorithm updates the centroids, adjusting their positions until each data point is closest to its assigned centroid, minimizing the overall distance within clusters.[2]. A detailed description of the methodology is given in Supplementary figure 1. |
| MOCAIP | The algorithm was developed to extract meaningful ICP-waveform features and uses also hierarchical clustering in two stages. Firstly, ICP-pulses are clustered and averaged (called dominant ICP-pulse). Subsequently, the dominant pulses are again clustered and compared to a reference library containing ICP-pulses in order to remove artefactual ICP-pulses [3]. |
| GBTM | Clusters individuals according to similar trajectories within a population, such as trajectories determined by age or time. Assessing the significance of these trajectories involves examining noticeable differences between groups in comparison to other clinical variables [4]. |
| Probabilistic graph model (Bayesian approach) | Åkerlund et al. performed analyses using probabilistic graph model Bayesian approaches. This method can handle a mix of data formats (discrete and continuous variables) and dealing with missing values [5]. In 2024, they expanded their approach by incorporating a Markov chain to account for the temporal aspect of the data[6]. |
| Spectral clustering | Clustering occurs through the utilization of a similarity matrix, which assigns weights to individual data points in comparison to others. It applies the graph Laplacian matrices and eigenvectors for spectral embedding (i.e., data reduction), and finally, employs a clustering algorithm such as k-means to group the data points in the set number of clusters. Note, other than with k-means, this method allows the identification of clusters with different data structures [7]. |
| Unsupersived Hidden Markov Models | The method approaches data as composed of various hidden (for example hourly) 'states'. It assumes that the observed data is influenced by an underlying process. To identify the underlying processes, probability functions are applied, and the optimization of these probabilities is carried out using an iterative process such as the EM-algorithm, as demonstrated by [8]. During the iterative optimization process, transition and prediction parameters are adjusted to maximize the likelihood of the different states. Note that each state transition depends only on the current state [9]. |
| Kohonen SOM | In essence, high-dimensional data is transformed through a process of iterative training, wherein each data point is mapped onto a two-dimensional grid of vectors (each vector matched the working dimension). This process involves identifying the datapoint with the closest matching vector, then the vector of the nearest and its neighbors' updates direction, and gradually converging the grid to reveal clusters, ultimately providing a simplified, visual representation for identifying patterns in two dimensions [10]. |
| EM = Expectation-Maximization; GBTM = Group-Based Trajectory Modeling; ICP = Intracranial Pressure; MOCAIP = Morphological Clustering and Analysis of Intracranial Pressure; SOM = Self-Organizing Maps. | |

| Supplementary table 4 \| Brief summary of feature Selection, clustering parameters, validation, and distance metrics (N= 18) | |
|---|---|
| Nelson et al, 2004 [10] | **Kohonen Self Organizing Maps (SOM)**<br>• **Feature selection:** input features defined by the authors. The features are eight MD-markers: glucose, lactate, pyruvate, and glutamate from both penumbral and non-penumbral tissues.<br>• **Number of nodes:** 900; the nodes define the dimension of the map into which the data is projected.<br>• **Deriving number of nodes:** the map size was derived by first allocating the first third period of MD-data to a map with different map sizes. These maps were then trained on data from the last third of the MD-period. The performance was measured by a comparison of both results by evaluating if datapoints were allocated to the same patient in both situations. This is expressed as a percentage correct prediction. The optimal separation is the size for which the percentage of correct prediction not changed by increasing map size.<br>• **Validation:** not explicitly reported.<br>• **Distance metrics:** Euclidean distance. |
| Haqqani et al., 2007 [11] | **K-means, Hierarchical**<br>• **Feature selection:** ICAT-based differential protein expression analysis using patient serum compared to pooled reference serum.<br>• **Number of clusters:** 16 proteins with a high pattern similarity to S100β or GCS.<br>• **Deriving number of clusters:** not explicitly reported.<br>• **Internal validation:** assessing different distance metrices.<br>• **Distance metrics:** Euclidean distance; Correlation distance-based method. |
| Sorani et al., 2007 [12] | **Hierarchical**<br>• **Feature selection:** input features were defined by the authors. The features were selected based on physiological variables captured through continuous monitoring in an ICU setting.<br>• **Number of clusters:** 3 clusters.<br>• **Deriving number of clusters:** the number of clusters was defined by the authors based on the hierarchical clustering results.<br>• **Validation:** not explicitly reported.<br>• **Distance metrics:** not explicitly reported. |
| Kim et al., 2011 [13] | **Morphological Clustering Analysis of ICP Pulse (MOCAIP) (includes hierarchical clustering)**<br>• **Feature selection:** the detection of ICP/CBFV pulses. Hierarchical clustering is used within the MOCAIP-algorithm to detect artifact-free pulses and ensure the quality of signal processing.<br>• **Number of clusters:** not explicitly reported.<br>• **Deriving number of clusters:** the Silhouette criterion is used to determine the optimal number of clusters [3].<br>• **Validation:** not explicitly reported.<br>• **Distance metrics:** Euclidean distance. |

| | |
|---|---|
| Wainwright et al., 2012 [14] | **Agglomerative hierarchical**<br>• **Feature selection:** inclusion of clinical data present in at least 65% of patients.<br>• **Number of clusters:** not applicable. The study did not define a specific number of clusters but instead used clustering to observe groupings of variables between survivors and non-survivors.<br>• **Deriving number of clusters:** not explicitly reported.<br>• **Validation:** not explicitly reported.<br>• **Distance metrics:** not explicitly reported. |
| Kumar et al., 2016 [15] | **K-means**<br>• **Dimensionality reduction:** PCA was applied to reduce the dimensionality of the CSF inflammatory markers before starting the clustering analysis.<br>• **Number of clusters:** 2 clusters.<br>• **Deriving number of clusters:** clustering was initially performed with five clusters. However, two clusters contained only one individual each, and another cluster with only four individuals was combined with a larger cluster due to similar mean principal component scores. This resulted in two final clusters for days 0-3.<br>• **Validation:** the cubic clustering criterion was applied to assess the quality of the cluster groups.<br>• **Distance metrics:** Euclidean distance. |
| Jha et al., 2018 [16] | **Group-Based Trajectory Modeling (GBTM)**<br>• **Feature selection:** the input features were predefined by the authors, focusing on longitudinal ICP measurements over a 120-hour period post-TBI, along with adjustments for various risk factors such as age, sex, initial GCS, craniectomy, and primary hemorrhage pattern.<br>• **Number of clusters:** six distinct ICP-trajectories<br>• **Deriving number of clusters:** the number of clusters was determined using the Bayesian Information Criterion. The model selection process involved iteratively eliminating non-significant polynomial terms to achieve the simplest final model where each trajectory group was significant at $\alpha$=0.05.<br>• **Validation:** not explicitly reported.<br>• **Distance metrics:** not applicable for this method, as GBTM is based on a probabilistic modeling approach rather than distance metrics. |
| Asgari et al., 2019 [8] | **Hidden Markov Models (HMM)**<br>• **Feature selection:** input features were defined by the authors and included hourly averaged values of ICP, CPP, RAP, and PRx.<br>• **Number of clusters:** three states (poor, intermediate and good state).<br>• **Deriving number of clusters:** pre-defined by the authors.<br>• **Internal validation:** assessment of the physiological relevance of the derived states such as assessing whether the states were statistically different in terms of the physiological variables, and whether the time spent in these states correlated with patient outcomes.<br>• **Distance metrics:** not applicable for this method, as HMM is a probabilistic model that does not rely on distance metrics. |

| | |
|---|---|
| Eiden et al., 2019 [17] | **Hierarchical**<br>• **Feature selection:** the input metabolites were selected based on the authors' criteria and supported by literature.<br>• **Number of clusters:** 2 metabolic states.<br>• **Deriving number of clusters:** the hierarchical tree's first branch divided the data into two clusters, interpreted as two distinct metabolic states.<br>• **External validation:** the findings were validated in a cohort of 12 patients.<br>• **Distance metrics:** not explicitly reported. |
| Gradisek et al., 2021 [18] | **Hierarchical**<br>• **Feature selection/ dimensionality reduction:** The LASSO method was used to select proteins associated with TBI-related variables such as the worst mGCS score. From the initial 107 biomarkers, 21 proteins were selected by three LASSO models, and 6 biomarkers (3 glial proteins and 3 cytokines) were ultimately identified as most relevant and used as input for the clustering analysis.<br>• **Number of clusters:** 3 clusters.<br>• **Deriving number of clusters:** not explicitly reported.<br>• **Validation:** not explicitly reported.<br>• **Distance metrics:** Euclidean distance. |
| Lindblad et al., 2021 [19] | **Hierarchical**<br>• **Feature selection/ dimensionality reduction:** the study calculated the CSF/serum ratio and included significant ratios for clustering. Specifically, proteins were included in the clustering analysis if the CSF/serum-ratio was significantly correlated with the albumin quotient, which is a marker of blood-brain barrier integrity.<br>• **Number of clusters:** 3 clusters in CSF; not explicitly reported for serum, but it can be deduced from Figure 3 that the authors found 2 clusters.<br>• **Deriving number of clusters:** selected hierarchical tree's branch as the number of clusters.<br>• **Validation:** validation was performed in a non-matched TBI cohort using serum samples only.<br>• **Distance metrics:** not explicitly reported. |
| Megjhani et al. 2021 [20] | **Morphological Clustering and Analysis of ICP (MOCAIP) (includes hierarchical clustering), K-means**<br>• **Feature selection:** hierarchical clustering is used within the MOCAIP-algorithm to detect artifact-free pulses [3].<br>• **Number of clusters:** k-means identified 20 clusters; hierarchical clustering results were not explicitly reported.<br>• **Deriving number of clusters:** not explicitly reported.<br>• **Validation:** not explicitly reported.<br>• **Distance metrics:** dynamic time warping was used as the distance metric for k-means clustering; Euclidean distance for hierarchical clustering [3]. |

| | |
|---|---|
| Narula et al., 2021 [21] | **Spectral clustering, k-means**<br>• **Feature selection:** EEG-data was split into 2-second windows, followed by the calculation of a covariance matrix for each window.<br>• **Number of clusters:** 2 clusters were identified, representing "burst" and "suppression".<br>• **Deriving number of clusters:** the authors explicitly defined 2 clusters (burst and suppression). Clustering was applied to each 2-second data window using the similarity matrix to segregate into these 2 clusters.<br>• **Validation:** the algorithm's performance was validated by comparing its results with ground truth annotations (manually labeled bursts and suppressions) and a supervised deep convolutional neural network.<br>• **Distance metrics:** Riemannian distance metric was used for the similarity matrix based on covariance matrices. |
| Åkerlund et al., 2022 [5] | **Probabilistic Graph Model (Bayesian Approach)**<br>• **Feature selection/ dimensionality reduction:** input features were defined by the authors. Based on clinical interest and known outcome predictors, particularly those with known or plausible relationships with outcome or deranged physiology.<br>• **Number of clusters:** 6 clusters.<br>• **Deriving number of clusters:** the number of clusters was determined using a stability-based approach [22]. Models with 3 to 15 clusters were created, and the optimal number was identified by evaluating the CSI across multiple iterations. The optimal clustering was defined as the number of clusters with the highest median CSI after adjusting for the number of clusters.<br>• **Internal validation:** internal stability was assessed by repeating the clustering process with different random initializations and evaluating the consistency of cluster assignments.<br>• **Distance metrics:** not applicable. The method used a mixture of probabilistic graph models that account for the relationships between features without relying on traditional distance metrics. |
| Boos et al, 2022 [23] | **K-means, Divisive/Agglomerative hierarchical**<br>• **Feature selection:** input features were defined by the authors, based on clinical and radiologic variables that had sufficient interrater reliability (k > 0.6).<br>• **Number of clusters:** 2 clusters.<br>• **Deriving number of clusters:** the optimal number of clusters (2 clusters) was determined using the Silhouette criterion and the gap statistic method for k-means. This number was used as the level in the hierarchical tree<br>• **Validation:** the authors compared the results produced by the three clustering algorithms and with the presence of a clinical "triad" to validate the robustness of the clusters.<br>• **Distance metrics:** Gower's method. |
| Rajagopalan et al., 2022 [24] | **Agglomerative hierarchical**<br>• **Feature selection:** input features were defined by the authors. The study included variables such as HR, MAP, ICP, brain tissue oxygenation, and cerebral MD (glucose, lactate, and pyruvate).<br>• **Number of clusters:** 4 clusters.<br>• **Deriving number of clusters:** the number of clusters was derived using the Caliński-Harabasz pseudo-F index. The index was calculated for groupings between 2 to 10 clusters, with the optimal number being 4 clusters.<br>• **Internal validation:** the stability of the clusters was assessed by comparing them with clusters derived using k-means clustering. |

| | |
|---|---|
| | • **Distance metrics:** Euclidean distance. |
| Satar et al., 2022 [25] | **K-means**<br>• **Feature selection:** the study calculated seven spectral features for the analysis: spectral centroid, spectral entropy, spectral flux, energy, energy entropy, zero crossing rate, and spectral roll-off.<br>• **Number of clusters:** 2 clusters.<br>• **Deriving number of clusters:** either hypoxic brain injury or no hypoxic brain injury.<br>• **Internal validation:** the clusters were validated using the Calinski-Harabasz index and the Rand index.<br>• **Distance metrics:** Euclidean distance. |
| Åkerlund et al. 2024 [6] | **Probabilistic Graph Model (Bayesian Approach) with a Markov chain extension**<br>• **Feature selection:** mutual information was computed to determine the importance of each feature in differentiating clusters. The most important features that best explained the differences between the patient groups (clusters) were then selected.<br>• **Number of clusters:** 6 clusters.<br>• **Deriving number of clusters:** the optimal number of clusters was identified using the EM-algorithm in combination with the CSI.<br>• **Validation:** internal stability was assessed by repeating the clustering process with different random initializations and evaluating the consistency of cluster assignments<br>• **Distance metrics:** not applicable for this method, as the method does not rely on a distance metrics. |

A brief summary of (I) study feature selection/dimensionality reduction methods. The feature selection can be defined by the authors, that means that the collected data is not prepared like computing or deriving measures before including the data in the clustering analysis; (II) the number of clusters, which were either configured before analyzing or set based on the results; (III) how the number of clusters were determined; (IV) which validation methods were applied both internal and/or external; (V) the distance metric, which is used to calculate the distance between data points and cluster centers. This is not required for every methodology.

CBFV = Cerebral Blood Flow Velocity; CPP = Cerebral Perfusion Pressure; CSF = Cerebrospinal Fluid

CSI = Cluster Similarity Index; CT = Computer Tomography; EM-algorithm = Expectation-Maximization Algorithm; GCS = Glasgow Coma Scale; GBTM = Group-Based Trajectory Modelling; HMM = Hidden Markov Models; HR = Heart Rate; ICAT-based = Isotope-Coded Affinity Tag ; ICP = Intracranial Pressure; ICU = Intensive Care Unit; LASSO = Least Absolute Shrinkage and Selection Operator ; MAP = Mean Arterial Blood Pressure MD = Microdialysis; MOCAIP = Morphological Clustering and Analysis of Intracranial Pressure; PCA = Principal Component Analysis; PRx = Pressure Reactivity Index; RAP = The correlation coefficient (R) between mean pulse amplitude (A) and mean intracranial pressure (P); SOM = Self-Organizing Maps. TBI = Traumatic Brain Injury.

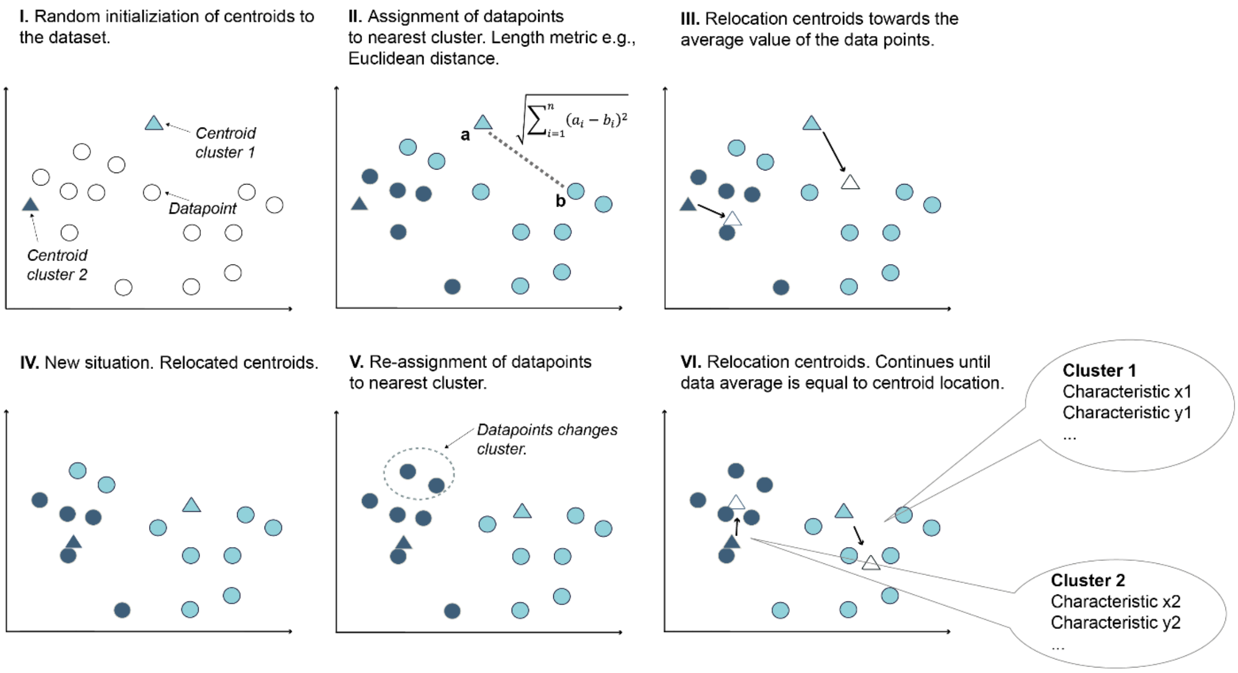


**Supplementary figure 1 |** Example of k-means algorithm. First, the datapoints are projected into an n-dimensional space where n depends on the number of input variables. Centroids (clustering centers) are randomly assigned to the dataset [26]. Datapoints are colored white, as they have not been yet assigned to a cluster (sub-figure I). In the next step, the distance between each datapoint and the centroid is computed. In the current example the Euclidean distance formula is used, but there are alternative distance metrics such as cosine distance, dot product etc.[27]. Once each datapoint has been assigned to a cluster (either cluster 1 or 2 in the current example), the centroids update its position to the average value within that cluster (sub-figure III). Due to the relocation of the centroids, the shortest distance to the nearest centroid changed for some datapoints (sub-figure IV and V). Therefore, the shortest distance between each datapoint and the centroids is computed again, resulting in two datapoints moving from cluster 2 to cluster 1 (sub-figure V). This iteration process (II – V) continues until the distance from each datapoint to a centroid is shortest and the centroids are the center of each cluster. The result is that the datapoints within each cluster are more related to each other than the datapoints between the clusters. This allows the characteristics of the patients within each cluster to be compared. For example, Cluster 1 includes patients with a low mortality and young age and cluster 2 patients with a high mortality and higher age. Various alternative models and parameter settings have been studied. The Figure is adapted from [28]. The methodologies applied in the included studies are briefly described in Supplementary file 1, table 3. The selected parameter settings within the studies are reported in Supplementary file 1, table 4.

**REFERENCES**


1. I. Kamber, Micheline. II. Pei JianIII (2012) Data mining : concepts and techniques, 3rd ed. Morgan Kaufmann Publishers is an imprint of Elsevier Inc.
2. Steinley D (2006) K-means clustering: a half-century synthesis. Br J Math Stat Psychol 59:1–34. https://doi.org/10.1348/000711005X48266
3. Hu X, Xu P, Scalzo F, Vespa P, Bergsneider M (2009) Morphological clustering and analysis of continuous intracranial pressure. IEEE Trans Biomed Eng 56:696–705. https://doi.org/10.1109/TBME.2008.2008636
4. Nagin DS, Odgers CL (2010) Group-based trajectory modeling in clinical research. Annu Rev Clin Psychol 6:109–138. https://doi.org/10.1146/annurev.clinpsy.121208.131413
5. Åkerlund CAI, Holst A, Stocchetti N, Steyerberg EW, Menon DK, Ercole A, Nelson DW, Investigat C-TP (2022) Clustering identifies endotypes of traumatic brain injury in an intensive care cohort: a CENTER-TBI study. Crit Care 26:228. https://doi.org/10.1186/s13054-022-04079-w
6. Åkerlund CAI, Holst A, Bhattacharyay S, Stocchetti N, Steyerberg E, Smielewski P, Menon DK, Ercole A, Nelson DW (2024) Clinical descriptors of disease trajectories in patients with traumatic brain injury in the intensive care unit (CENTER-TBI): a multicentre observational cohort study. Lancet Neurol 23:71–80. https://doi.org/10.1016/S1474-4422(23)00358-7
7. von Luxburg U (2007) A tutorial on spectral clustering. Stat Comput 17:395–416. https://doi.org/10.1007/s11222-007-9033-z
8. Asgari S, Adams H, Kasprowicz M, Czosnyka M, Smielewski P, Ercole A (2019) Feasibility of Hidden Markov Models for the Description of Time-Varying Physiologic State After Severe Traumatic Brain Injury. Crit Care Med 47:E880–E885. https://doi.org/10.1097/CCM.0000000000003966
9. Franzese M, Iuliano A (2019) Hidden Markov Models. In: Ranganathan S, Gribskov M, Nakai K, Schönbach C (eds) Encyclopedia of Bioinformatics and Computational Biology. Academic Press, Oxford, pp 753–762
10. Nelson DW, Bellander B-M, Maccallum RM, Axelsson J, Alm M, Wallin M, Weitzberg E, Rudehill A (2004) Cerebral microdialysis of patients with severe traumatic brain injury exhibits highly individualistic patterns as visualized by cluster analysis with self-organizing maps. Crit Care Med 32:2428–2436. https://doi.org/10.1097/01.ccm.0000147688.08813.9c
11. Haqqani AS, Hutchison JS, Ward R, Stanimirovic DB, Translational CCC (2007) Protein biomarkers in serum of pediatric patients with severe traumatic brain injury identified by ICAT-LC-MS/MS. J Neurotrauma 24:54–74. https://doi.org/10.1089/neu.2006.0079
12. Sorani MD, Hempbill III JC, Morabito D, Rosenthal G, Manley GT (2007) New approaches to physiological informatics in neurocritical care. Neurocrit Care 7:45–52. https://doi.org/10.1007/s12028-007-0043-7
13. Kim S, Hu X, McArthur D, Hamilton R, Bergsneider M, Glenn T, Martin N, Vespa P (2011) Inter-subject correlation exists between morphological metrics of cerebral blood flow velocity and intracranial pressure pulses. Neurocrit Care 14:229 – 237. https://doi.org/10.1007/s12028-010-9471-x
14. Wainwright MS, Lewandowski R (2012) Bioinformatics analysis of mortality associated with elevated intracranial pressure in children. Acta Neurochir Suppl 114:67–73. https://doi.org/10.1007/978-3-7091-0956-4_12
15. Kumar RG, Rubin JE, Berger RP, Kochanek PM, Wagner AK (2016) Principal components derived from CSF inflammatory profiles predict outcome in survivors after severe traumatic brain injury. Brain Behav Immun 53:183–193. https://doi.org/10.1016/j.bbi.2015.12.008
16. Jha RM, Elmer J, Zusman BE, Desai S, Puccio AM, Okonkwo DO, Park SY, Shutter LA, Wallisch JS, Conley YP, Kochanek PM (2018) Intracranial Pressure Trajectories: A Novel Approach to Informing Severe Traumatic Brain Injury Phenotypes. Crit Care Med 46:1792–1802. https://doi.org/10.1097/CCM.0000000000003361
17. Eiden M, Christinat N, Chakrabarti A, Sonnay S, Miroz J-P, Cuenoud B, Oddo M, Masoodi M (2019) Discovery and validation of temporal patterns involved in human brain ketometabolism in cerebral

microdialysis fluids of traumatic brain injury patients. EBioMedicine 44:607–617. https://doi.org/10.1016/j.ebiom.2019.05.054

18. Gradisek P, Carrara G, Antiga L, Bottazzi B, Chieregato A, Csomos A, Fainardi E, Filekovic S, Fleming J, Hadjisavvas A, Kaps R, Kyprianou T, Latini R, Lazar I, Masson S, Mikaszewska-Sokolewicz M, Novelli D, Paci G, Xirouchaki N, Zanier E, Nattino G, Bertolini G (2021) Prognostic Value of a Combination of Circulating Biomarkers in Critically Ill Patients with Traumatic Brain Injury: Results from the European CREACTIVE Study. J Neurotrauma 38:2667–2676. https://doi.org/10.1089/neu.2021.0066

19. Lindblad C, Pin E, Just D, Al Nimer F, Nilsson P, Bellander B-M, Svensson M, Piehl F, Thelin EP (2021) Fluid proteomics of CSF and serum reveal important neuroinflammatory proteins in blood-brain barrier disruption and outcome prediction following severe traumatic brain injury: a prospective, observational study. Crit Care 25:. https://doi.org/10.1186/s13054-021-03503-x

20. Megjhani M, Terilli K, Kaplan A, Wallace BK, Alkhachroum A, Hu X, Park S (2021) Use of Clustering to Investigate Changes in Intracranial Pressure Waveform Morphology in Patients with Ventriculitis. Acta Neurochir Suppl 131:59–62. https://doi.org/10.1007/978-3-030-59436-7_13

21. Narula G, Haeberlin M, Balsiger J, Strässle C, Imbach LL, Keller E (2021) Detection of EEG burst-suppression in neurocritical care patients using an unsupervised machine learning algorithm. Clinical Neurophysiology 132:2485 – 2492. https://doi.org/10.1016/j.clinph.2021.07.018

22. Lange T, Roth V, Braun ML, Buhmann JM (2004) Stability-based validation of clustering solutions. Neural Comput 16:1299–1323. https://doi.org/10.1162/089976604773717621

23. Boos SC, Wang M, Karst WA, Hymel KP, Pe PBIRN (2022) Traumatic Head Injury and the Diagnosis of Abuse: A Cluster Analysis. Pediatrics 149:. https://doi.org/10.1542/peds.2021-051742

24. Rajagopalan S, Baker W, Mahanna-Gabrielli E, Kofke AW, Balu R (2022) Hierarchical Cluster Analysis Identifies Distinct Physiological States After Acute Brain Injury. Neurocrit Care 36:630–639. https://doi.org/10.1007/s12028-021-01362-6

25. Satar M, Cengizler C, Hamitoglu S, Ozdemir M (2022) Investigation of Relation Between Hypoxic-Ischemic Encephalopathy and Spectral Features of Infant Cry Audio. Journal of Voice. https://doi.org/10.1016/j.jvoice.2022.05.015

26. Trevor Hastie, Robert Tibshirani JF (2009) The Elements of Statistical Learning: Data Mining, Inference, and Prediction, Second Edition, 2nd ed. Springer New York, NY, NY

27. Gao CX, Dwyer D, Zhu Y, Smith CL, Du L, Filia KM, Bayer J, Menssink JM, Wang T, Bergmeir C, Wood S, Cotton SM (2023) An overview of clustering methods with guidelines for application in mental health research. Psychiatry Res 327:115265. https://doi.org/https://doi.org/10.1016/j.psychres.2023.115265

28. Clustering 2: soft vs. hard clustering Clustering 2: soft vs. hard clustering . Accessed Juni 2024, https://www.youtube.com/watch?v=_aWzGGNrcic
